# Association Between Intellectual Functioning and Autistic Traits in the General Population of Children

**DOI:** 10.1007/s10578-023-01562-5

**Published:** 2023-06-23

**Authors:** Maria Marinopoulou, Eva Billstedt, Catrin Wessman, Carl-Gustaf Bornehag, Maria Unenge Hallerbäck

**Affiliations:** 1 Gillberg Neuropsychiatry Centre, Institute of Neuroscience and Physiology, Sahlgrenska Academy, University of Gothenburg, Gothenburg, Sweden; 2 Child and Adolescent Habilitation, Region Värmland, Karlstad, Sweden; 3 Child Neuropsychiatric Clinic, Sahlgrenska University Hospital, Gothenburg, Sweden; 4 School of Public Health and Community Medicine, Institute of Medicine, University of Gothenburg, Gothenburg, Sweden; 5 Department of Health Sciences, Karlstad University, Karlstad, Sweden; 6 Icahn School of Medicine at Mount Sinai, New York, NY USA; 7 School of Medical Sciences, Faculty of Medicine and Health, Örebro University, Örebro, Sweden

**Keywords:** Intellectual functioning, Autistic traits, Behavioural problems, Wechsler scales

## Abstract

Autistic traits are continuously distributed in the general population. The associations between autistic traits and intellectual functioning and/or behavioural difficulties, and the impact of intellectual functioning on behavioural difficulties are unclear. The study aims to describe the distribution of autistic traits in a population-based cross-sectional sample of children. Further aims are to examine the association between intellectual functioning and autistic traits, and between autistic traits and behavioural difficulties. Wechsler scales and ratings of autistic traits and behavioural problems in 874 children aged 7–9 years in the Swedish Environmental Longitudinal Mother and Child, Asthma and Allergy (SELMA) study were assessed. We found a continuous distribution of autistic traits. Intellectual functioning was negatively associated with autistic traits but not with behavioural difficulties. Behavioural difficulties were associated with autistic traits.

Autism spectrum disorder (ASD) is a neurodevelopmental disorder characterized by deficits in social communication and social interaction, and restrictive, repetitive behaviours and interests [1]. ASD has a prevalence of about 1.5% [2], where boys are more often affected. A recent meta-analysis has reported a male-to-female ratio of about 3:1 [3]. Several studies in population-based samples of children have found that autistic traits are continuously distributed in the general population, i.e., they are not limited to children with ASD diagnosis and are present on a continuum ranging from children with virtually no autistic traits to children with clinical diagnosis of ASD [4–7]. ASD is the extreme end of this continuum [6, 8, 9] and ASD and autistic traits share genetic and biological aetiology [8, 9]. Only one study, as far as we know, has particularly focused on the distribution of autistic traits in children in the first school years, i.e., aged 7–8 years [4]. Even though ASD is possible to diagnose in preschool years, diagnosis at later age is common, illustrating the need for earlier detection [10–12] in order to provide intervention [13, 14].

Intelligence, which is important for academic outcomes in the general population [15], has been associated with ASD. In ASD, childhood intelligence quotient (IQ) is a strong predictor of later outcome. Higher childhood IQ is associated with less severe autism symptoms and better adaptive functioning in childhood [16–18]. Furthermore, there seems to be an association between higher IQ and ability to compensate for difficulties in cognitive skills, e.g., theory of mind, in ASD [19]. In earlier studies, about 70% of the children with ASD showed intellectual disability (ID) [20, 21]. With broader definition and higher prevalence of ASD, the proportion of children with an IQ below 70 has diminished [22–24]. Recent studies have reported that about 35–47% of children and adolescents with ASD have IQ ≤ 70 [25, 26], thus indicating that nearly half of the children with ASD or more have IQ > 70. This means that a majority of children with ASD attends mainstream schools.

Some studies have investigated the relationship between intellectual functioning and autistic traits. In studies of clinical samples of children with ASD and using the Social Responsiveness Scale (SRS) as measure of autistic traits, not completely consistent results have been reported. Constantino et al. (2003) found no relation between autistic traits and IQ in a small clinical sample of children, whereas Bölte et al. (2008) found little to negligible correlations between the SRS and IQ in a clinical sample of 527 children and adolescents with a mean age of 10.3 years. In a sample of both clinical and typically developing children, aged 4–18 years, no correlation was found between SRS scores and IQ as regards children with IQ > 70, but children with ID tended to receive higher SRS scores [5]. Further instruments have been used in order to study the connection between autistic traits and IQ. The Child Autism Spectrum Test [27] has been used in population-based twin studies [28, 29], that have included measurements during childhood and IQ measured by selected subtests and/or tests administered through telephone, booklets, and web-based. The results showed a modest negative association between extreme autistic traits, and low IQ [28], and between autistic traits and the full-range of IQ scores [29]. The Autism Spectrum Screening Questionnaire (ASSQ [30]) was used by Ryland et al. [31] to examine the associations between IQ, sex, and autistic traits in a sample of 325 children aged 8–12 years drawn from a population-based cohort. The authors also examined associations between verbal IQ and performance IQ discrepancy and autistic traits. They reported that IQ and the majority of ASSQ scores correlated negatively regardless of sex. ASSQ scores declined with higher IQ. Displaying a significant discrepancy between verbal and performance IQ was mostly not linked to ASSQ scores.

As regards additional difficulties, autistic traits, even of lower degree, have been associated with risk for Attention Deficit Hyperactivity Disorder (ADHD), anxiety and conduct problems [32]. In studies using the Strengths and Difficulties Questionnaire (SDQ), a brief screening of emotional and behavioural problems [33], autistic traits have also been associated with internalizing problems [34, 35] and peer problems later on in childhood [35]. Children with ASD, both in population-based samples and samples recruited from clinics, have been reported to have more problems measured with SDQ than controls [36, 37].

Taken together, population-based studies exploring the relationship between intellectual functioning and autistic traits in children render mixed results. This could be due to the inclusion of clinical vs. non-clinical samples, different age groups, and the use of different instruments to measure IQ as well as autistic traits. It might also be so that the association between IQ and autistic traits differs between groups with different degree of autistic traits. The SRS is a well-validated instrument measuring autistic traits suitable for both clinical and non-clinical populations, and providing sex-specific scores [38], which decreases the risk of missing female cases in diagnostics. The Wechsler scales administered by psychologists are the most frequently used instrument to measure intellectual functioning [39], and their clinical utility has been demonstrated in several settings [40]. Examining the relationship between autistic traits and intellectual functioning in a narrow age range and a large sample from the general population could further clarify these associations.

## Aim

The aims of the present study are to: (a) describe the distribution of autistic traits in a population-based sample of 7–9 year old children, (b) examine the association between intellectual functioning and autistic traits, and (c) between autistic traits and behavioural difficulties.

## Methods

### Participants and Procedure

The sample includes children from the Swedish Environmental Longitudinal Mother and Child, Asthma and Allergy (SELMA) study, in Värmland County, Sweden [41]. The SELMA study is a population-based pregnancy cohort study with the primary aim to examine prenatal exposure for environmental factors and the effects on children´s health and development. SELMA recruited approximately 2,300 pregnant women between September 2007 and March 2010, and, after taking into account drop out because of miscarriages and other pregnancy-related circumstances, 1,954 children were born. Detailed description of the recruitment process has been provided in Bornehag et al. [41]. At age 7, 1,006 children went through a neuropsychological assessment. The assessments were performed between September 2015 and August 2018 by licensed psychologists and in a few cases by psychologists in training under supervision. Inclusion in the study required that children had participated in test assessment and their parents had completed both questionnaires included in the study, in total 894 children. However, 17 children had uncompleted tests and parental questionnaire was uncompleted for 3 children. The final sample consists of 874 children (444 boys, 430 girls) with data on the psychological test and questionnaires used in the study. The mean age was 7.9 years (SD 0.5 years, range 7.0-9.8 years), with no significant age difference between boys and girls. About 68% of the mothers had completed college or university studies. For 866 of the children, there are data on the presence or absence of clinical ASD diagnosis, collected with parental interview.

### Measures

#### The Wechsler Intelligence Scale for Children – Fourth Edition (WISC-IV)

The WISC-IV [42] is a psychological test with strong psychometrics properties used for assessment of cognitive function in children at ages 6–16 years. The WISC-IV includes 10 core and five supplemental subtests. In addition to a score of general intellectual functioning (Full Scale IQ, FSIQ), WISC-IV provides four index scores [Verbal Comprehension Index (VCI), Perceptual Reasoning Index (PRI), Working Memory Index (WMI), and Processing Speed Index (PSI)], and a composite score of General Ability Index (GAI) [42]. The mean and SD for FSIQ and the indices are 100 and 15, respectively. The average reliability coefficients for FSIQ (0.97) and the indices (0.88-0.94) are high, and the WISC-IV correlates substantially with other global measures [40]. In the study, the Swedish version of WISC-IV was used to evaluate cognitive function.

#### The Social Responsiveness Scale (SRS)

The parental version of the SRS [38] was used to measure autistic traits in the sample. The SRS is a well-validated quantitative questionnaire measuring autistic traits in children aged 4–18 years. The SRS comprises of 65 items, rating the frequency of a particular behaviour, in the domains of social communication and social interaction, and restrictive, repetitive behaviours and interests. Raw scores are converted to T-Scores, i.e., standardized scores scaled on a distribution with a mean of 50 and SD of 10. Because of sex-differences, the manual provides sex-based references. Results generate a standardized total score (SRS Total T-Score, range 34–127 for boys and 38–110 for girls). Higher T-Scores indicate more impairment and greater degree of symptoms. T-Scores of 60 through 75 are in the mild to moderate range, while T-Scores of 76 or higher are in the severe range. Both indicate clinically significant deficits that interfere with social interactions in daily life, but 76 or higher indicate a severe interference [38]. The SRS raw scores also generate five subscales: Social Awareness, Social Cognition, Social Communication, Social Motivation and Autistic Mannerisms, whose scores can be converted to T-Scores. However, the subscales lack cut-offs since they are not used for screening or diagnostic purposes. Parental report with the SRS correlated very well with the Autism Diagnostic Interview-Revised (ADI-R), which is considered a gold standard for ASD assessment [43]. The cross-cultural validity of the SRS has been demonstrated in a large European sample [44]. The internal consistency of the parent rated SRS total scores reported in the manual is good, alpha = 0.94 in males and 0.93 in females [38], similar to those found in a UK sample from the general population [45] and the normative sample in the cross-cultural validation study [44]. In our sample, the Cronbach alpha coefficient for the total SRS was very good (0.92). The internal consistency for the subscales is good (0.76-0.82), except for Social Awareness (0.51) and Social motivation (0.64), which resembles the varied alpha coefficients for subscales in [45]. The subscales are not used in our study since they are for clinical purposes (treatment planning and effectiveness) [38].

#### The Strengths and Difficulties Questionnaire (SDQ)

The SDQ [33] is a child and adolescent behaviour screening instrument comprising 25 items. Answers are given as “Not true”, “Somewhat true”, or “Certainly true”, and are transformed into values 0–2, depending on the item. The items are used to calculate five scales with five items each: Emotional problems, Conduct problems, Hyperactivity/Inattention, Peer relationship problems and Prosocial behaviour (score range 0–10 for each scale). The sum of the scales, excluding Prosocial behaviour, provides a total difficulties score (SDQ Total Difficulties Score, range 0–40, higher score indicates more problems). The Swedish cut-off according to 90th percentile for the SDQ Total Difficulties Score is 14 [46]. The psychometric properties of the SDQ are satisfactory to good [47, 48], and the Swedish parental version used in this study has been validated in 5-15-year-olds in Sweden [46, 49]. In our sample, the Cronbach alpha coefficient for the SDQ Total Difficulties was good (0.79). The Hyperactivity/Inattention scale showed the highest internal consistency (0.81), followed by moderate coefficients for Prosocial behaviour (0.68) and Emotional problems (0.63). Peer problems (0.51) and Conduct problems (0.53) had the lowest internal consistency coefficients, consistent with Essau et al. [47].

### Statistical Analysis

Mean and median of the SRS and the SDQ scores and mean of the WISC-IV Index scores were computed for the sample and both sexes. Differences in the mean SRS Total Raw between sexes and differences in the SRS Total T-Scores between sexes were examined with independent samples t-tests. To describe the distribution of autistic traits in the sample, frequencies and cumulative percentages were calculated for the SRS Total T-Score. The percentage of children meeting the SRS Total T-Score cut-off scores was calculated. Chi-square test of independence was used to compare the proportion of boys and girls scoring below and at/above SRS cut-offs. The relationship between the WISC-IV index scores and the SRS Total T-Score was explored with Pearson correlation. Quantile regression was used to estimate: (a) the impact of intellectual functioning (FSIQ), age, and sex on autistic traits (SRS Total T-Score) at each quartile (25%, 50%, and 75% respectively) of SRS Total T-Score; (b) the impact of WISC-IV index scores, age, and sex on autistic traits (SRS Total T-Score ) at each quartile (25%, 50%, and 75% respectively) of SRS Total T-Score. These two analyses were performed to examine the association between intellectual functioning and autistic traits. Furthermore, to examine the association between autistic traits and behavioural difficulties, we used quantile regression to estimate the impact of autistic traits (SRS Total T-Score), intellectual functioning (FSIQ), age, and sex on behavioural difficulties (SDQ Total Difficulties Score) at each quartile (25%, 50%, and 75% respectively) of SDQ Total Difficulties Score. Since data is score data and skewed (and hence, median is a more suitable measure), we chose quantile regression to achieve a comprehensive analysis of these relationships. Quantile regression allows us to estimate different quantiles, using the response as a continuous variable [50]. This is a powerful tool, when data is skewed or the assumptions of linear regression are not fulfilled. Just in a similar way as linear regression is estimating the mean effect, quantile regression estimates for instance the median effect (or either other quantile). It might also be that different covariates have different effects for lower quantiles compared to higher quantiles in the response spectra. The sample was divided in 3 groups based on the SRS Total T-Score and mean SDQ scores were calculated for the groups. IBM SPSS for Windows version 27 was used for all analyses, except for the quantile regressions that were performed with R Studio. The statistical significance threshold was set at p < .05. We did not adjust for multiplicity.

## Results

### WISC-IV and SDQ Results

The mean WISC-IV Index scores of the participants were in the average range, with the exception of WMI that was in the low average range (Table 1).

**Table 1:** WISC-IV Index Scores, SRS T-Scores and SDQ Scores for the total sample, boys and girls

	Total sample N = 874			Boys n = 444			Girls n = 430		
	Mean	SD		Mean	SD		Mean	SD	
WISC-IV									
Full Scale IQ	99.9	12.7		98.2	12.6		101.6	12.6	
Verbal Comprehension	100.7	11.4		99.7	11.6		101.7	11.2	
Perceptual Reasoning	106.2	12.4		105.9	12.5		106.6	12.3	
Working Memory	89.9	13.6		88.9	13.4		91.0	13.8	
Processing Speed	98.9	15.2		95.8	15.2		102.0	14.7	
SRS	Mean	SD	Median	Mean	SD	Median	Mean	SD	Median
Total T-Score	45.9	8.3	44.0	45.3	8.3	43.0	46.4	8.3	45.0
SDQ									
Total Difficulties Score	5.8	4.6	5.0	6.1	4.7	5.0	5.5	4.5	4.0
Emotional problems	1.5	1.7	1.0	1.4	1.6	1.0	1.6	1.8	1.0
Conduct problems	1.1	1.4	1.0	1.1	1.3	1.0	1.1	1.4	1.0
Hyperactivity/Inattention	2.5	2.4	2.0	2.9	2.4	3.0	2.1	2.2	1.0
Peer problems	0.7	1.2	0.0	0.7	1.2	0.0	0.6	1.1	0.0
Prosocial behaviour ^a^	8.8	1.5	9.0	8.6	1.6	9.0	9.0	1.4	10.0

^a^ Higher scores indicate more prosocial behaviour

Eight children in the sample (0.9%) had a FSIQ score of ≤ 70, indicative of possible ID. Of those, only 2 had a diagnosis of ID, as reported in the parental interview. Fourteen children in the sample (1.6%) had a diagnosis of ASD. Mean and median SDQ scores for the sample, boys, and girls are reported in Table 1.

### Distribution of Autistic Traits in the Sample

The mean SRS Total Raw Score in the sample was 22.6 (SD = 16.3). There was a significant difference between the mean SRS Total Raw Scores for boys (M = 23.9, SD = 17.3) and girls (M = 21.2, SD = 15.0); t(872) = 2.52, *p* = .01, *d* = 0.170. After conversion of raw scores to T-Scores, that are sex-normed, girls had marginally higher mean SRS Total T-Score (M = 46.3, SD = 8.3) than boys (M = 45.3, SD = 8.3); t(872)=-1.99, *p* = .046, *d*=-0.135. Table 1 shows the mean and median of the SRS Total T-Score for the total sample, boys, and girls.

**Fig. 1:**
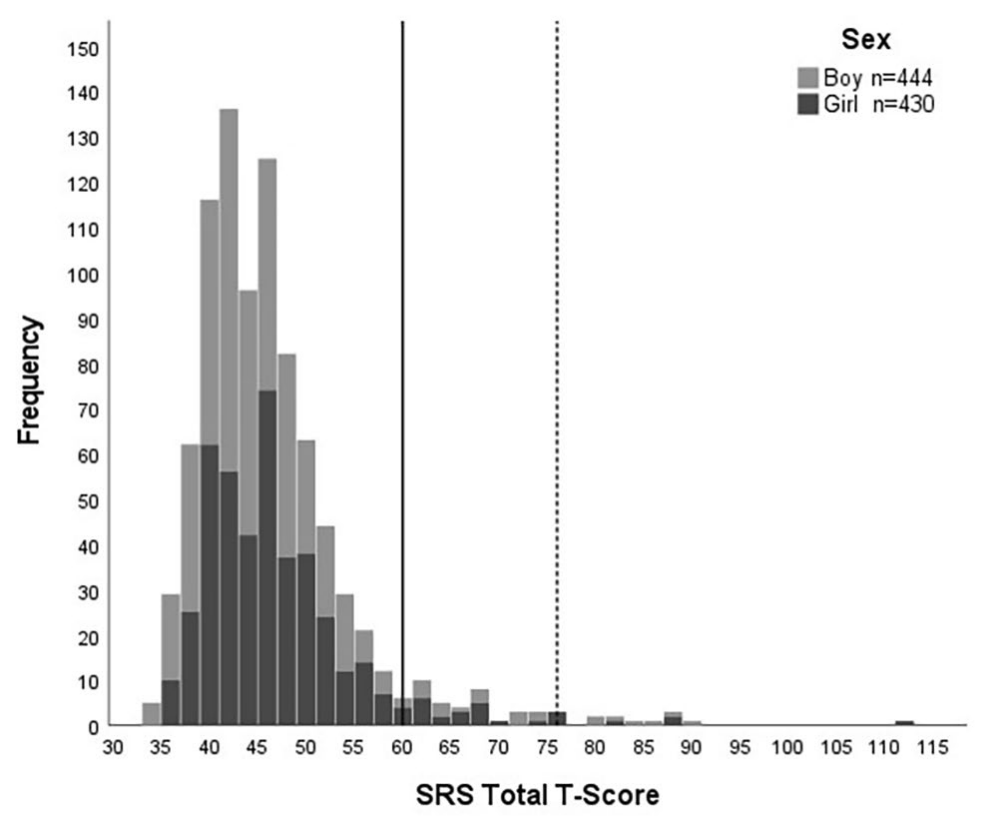
Distribution of autistic traits (SRS Total T-Score) by sex *Note*. Solid line shows the cut-off T-Score of 60 and dashed line shows the cut-off T-Score of 76

The distribution of autistic traits in the sample measured with SRS Total T-Score is presented in Fig. 1. Table 2 shows the frequency, percentage and cumulative percentage of SRS Total *T*-Scores in the sample.

**Table 2:** Frequency, percentage, and cumulative percentage of SRS Total T-Scores in the sample (N = 874)

SRS Total T-Score	Frequency	Percent	Cumulative percent
34–35	16	1.8	1.8
36	18	2.1	3.9
37	24	2.7	6.6
38	38	4.3	11.0
39	52	5.9	16.9
40	64	7.3	24.3
41	56	6.4	30.7
42	80	9.2	39.8
43	46	5.3	45.1
44	50	5.7	50.8
45	64	7.3	58.1
46	61	7.0	65.1
47	53	6.1	71.2
48	29	3.3	74.5
49	38	4.3	78.8
50	25	2.9	81.7
51	18	2.1	83.8
52	26	3.0	86.7
53	19	2.2	88.9
54	10	1.1	90.0
55	10	1.1	91.2
56	11	1.3	92.4
57	8	0.9	93.4
58	4	0.5	93.8
59	6	0.7	94.5
61	7	0.8	95.3
62	3	0.3	95.7
63	4	0.5	96.1
64	1	0.1	96.2
65	3	0.3	96.6
66	1	0.1	96.7
67	5	0.6	97.3
68	3	0.3	97.6
70	1	0.1	97.7
71	2	0.2	97.9
72	1	0.1	98.1
74	3	0.3	98.4
75	2	0.2	98.6
≥ 76	12	1.4	100
Total	874	100	

Forty-eight children (5.5%) had a SRS Total T-Score of 60 or higher indicating scores within clinical range according to the SRS manual [38]. Of those, 36 (4.1%, 16 boys, 20 girls) had a T-Score between 60 and 75 corresponding to the mild to moderate range of SRS, and 12 (1.4%, 7 boys, 5 girls) had a T-Score of 76 or higher corresponding to the severe range. The proportion of boys and girls scoring below cut-off, in the range between T-Scores 60 and 75, and at T-Scores of 76 or higher did not differ (*X*^2^ (2, *N* = 874) = 0.86, *p* = .65). The majority of the children with a previous ASD diagnosis (12 of 14) scored at 70 or higher.

### Association Between Intellectual Functioning and Autistic Traits

SRS Total T-Score correlated negatively with FSIQ (*r*=-.24, *p* < .001), VCI (*r*=-.27, *p* < .001), PRI (*r*=-.19, *p* < .001), WMI (*r*=-.21, *p* < .001), and PSI (*r*=-.07, *p* = .04) respectively.

**Fig. 2:**
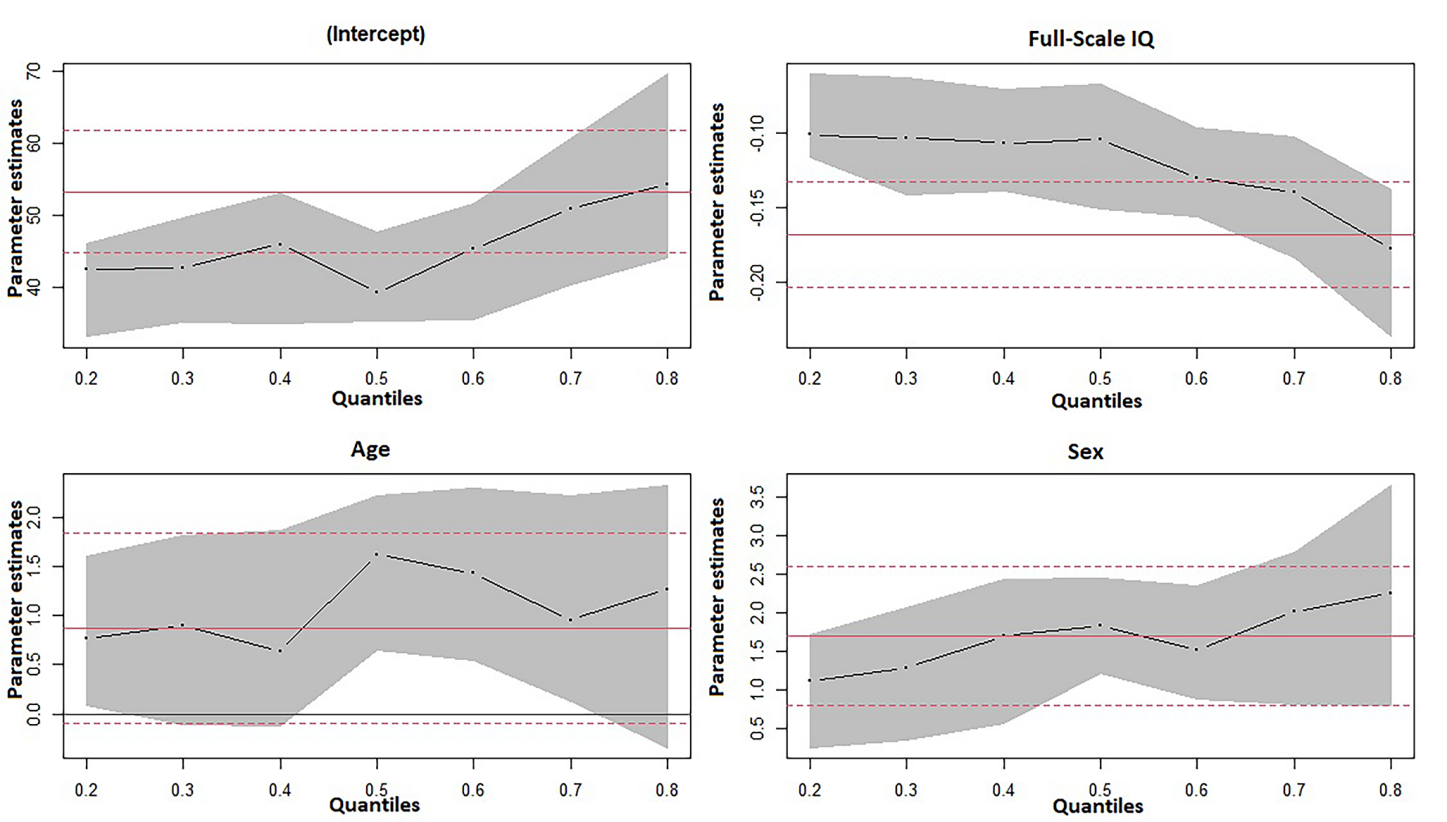
Parameter estimates for the intercept, Full Scale IQ, age and sex, respectively, at various quantiles of the SRS Total T-Score distribution *Note*. The horizontal solid red lines present Ordinary Least Squares (OLS) parameter estimates with 95% confidence intervals (dashed red lines)

In the first quantile regression model, intellectual functioning (FSIQ) and sex were significantly associated with autistic traits (SRS Total T-Score) throughout all percentiles of the SRS Total T-Score distribution (Fig. 2; Table 3). However, they were associated somewhat differently across the lower and the upper ends of the SRS Total T-Score distribution. Age was associated with autistic traits only around the 50th percentile, but not in the 25th and 75th percentile.

In the second quantile regression model using the WISC-IV indices, VCI and WMI were negatively associated with autistic traits (SRS Total T-Score) throughout all percentiles of the SRS Total T-Score distribution (Table 3). VCI was differently associated across the lower and upper ends of the SRS Total T-Score distribution. In this model, association between age and autistic traits was only around the 50th percentile. Sex was associated with autistic traits, but not in the lower ends. PRI and PSI were not associated with autistic traits.

**Table 3:** Results of quantile regression analyses estimating (1) the impact of intellectual functioning (Full Scale IQ), age and sex on autistic traits at different quantiles of the SRS Total T-Score, and (2) the impact of WISC-IV index scores, age, and sex on autistic traits (SRS Total T-Score) at every 0.25 quantile of SRS Total T-Score

SRS Total T-Score									
	Q (0.25)		Q (0.5)			Q (0.75)			
	Coefficients (95%CI)	*p*	Coefficients (95%CI)		*p*	Coefficients (95%CI)		*p*	
Model 1									
Intercept	43.36 (36.45, 49.35)	< 0.001	39.26 (35.37, 47.65)		< 0.001	50.38 (40.39, 64.70)		< 0.001	
Full Scale IQ	-0.10 (-0.13, -0.07)	< 0.001	-0.10 (-0.15, -0.07)		< 0.001	-0.17 (-0.21, -0.11)		< 0.001	
Age	0.77 (-0.03, 1.53)	0.108	1.62 (0.65, 2.22)		0.006	1.49 (-0.22, 2.51)		0.065	
Sex	1.02 (0.39, 1.76)	0.015	1.84 (1.22, 2.46)		< 0.001	2.32 (0.87, 3.31)		0.001	
Model 2									
Intercept	47.10 (37.61, 51.90)	< 0.001	45.85 (38.77, 59.60)		< 0.001	58.70 (51.56, 71.73)		< 0.001	
Verbal Comprehension	-0.07 (-0.11, -0.04)	0.004	-0.10 (-0.16, -0.05)		0.006	-0.17 (-0.23, -0.14)		< 0.001	
Perceptual Reasoning	-0.03 (-0.06, 0.01)	0.151	-0.01 (-0.06, 0.05)		0.867	0.03 (-0.02, 0.07)		0.364	
Working Memory	-0.05 (-0.07, -0.02)	0.001	-0.06 (-010, -0.02)		0.007	-0.07 (-0.10, -0.02)		0.026	
Processing Speed	0.02 (-0.01, 0,04)	0.295	0.01 (-0.02, 0.03)		0.704	0.00 (-0.03, 0.03)		0.946	
Age	0.72 (0.09, 1.70)	0.154	1.42 (0.11, 2.09)		0.020	0.95 (-0.24, 1.95)		0.202	
Sex	0.75 (0.25, 1.54)	0.062	1.60 (0.88, 2.32)		0.001	1.75 (0.96, 2.56)		0.002	

### Association Between Autistic Traits and Behavioural Difficulties

**Fig. 3:**
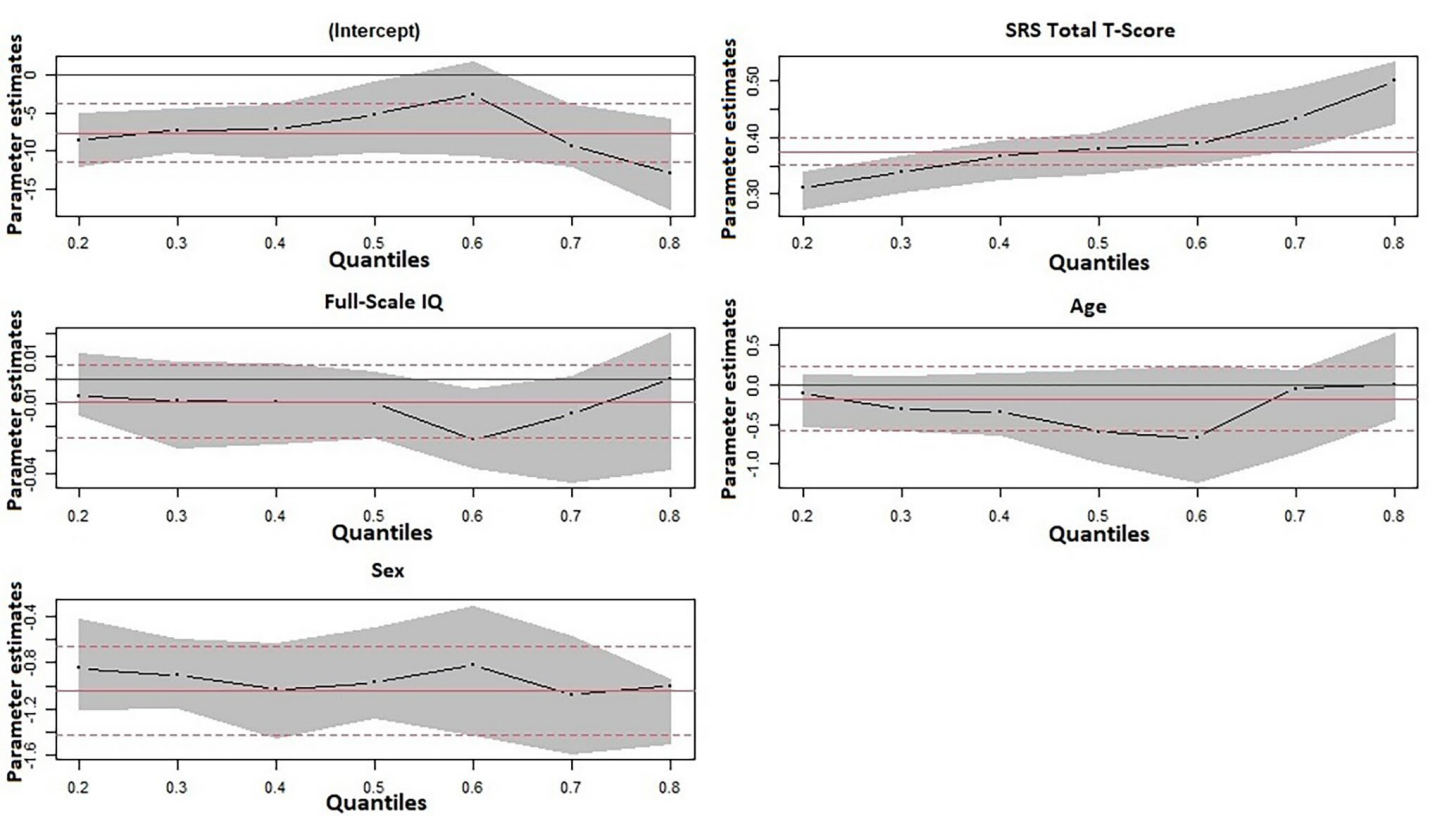
Parameter estimates for the intercept, SRS Total T-Score, Full Scale IQ, age and sex, respectively, at various quantiles of the SDQ Total Difficulties Score distribution *Note*. The horizontal solid red lines present Ordinary Least Squares (OLS) parameter estimates with 95% confidence intervals (dashed red lines)

As displayed in Fig. 3; Table 4, autistic traits (SRS Total T-Score) and sex were significantly associated with behavioural difficulties (SDQ Total Difficulties Score) throughout all percentiles of the SDQ Total Difficulties Score distribution. SRS Total T-Score was positively but differently associated with behavioural difficulties when comparing the lower and the upper percentiles of the SDQ Total Difficulties Score distribution, i.e., those scoring higher in SDQ Total Difficulties had more autistic traits. Being a girl was associated with fewer points in SDQ Total Difficulties Score, and this was the case both for those who scored lower and for those who scored higher in the SDQ Total Difficulties Score. FSIQ and age were not associated with behavioural difficulties (SDQ Total Difficulties Score).

**Table 4:** Results of quantile regression estimating the impact of autistic traits (SRS Total T-Score), intellectual functioning (Full Scale IQ), age and sex on behavioural difficulties at different quantiles of the SDQ Total Difficulties Score

SDQ Total Difficulties Score									
	Q(0.25)		Q(0.5)			Q(0.75)			
	Coefficients (95%CI)	*p*	Coefficients (95%CI)		*p*	Coefficients (95%CI)		*p*	
Intercept	-7.83 (-10.37, -4.40)	< 0.001	-5.14 (-10.15, -0.85)		0.073	-7.57 (-13.51, -2.05)		0.045	
SRS Total T-Score	0.32 (0.30, 0.35)	< 0.001	0.38 (0.34, 0.41)		< 0.001	0.46 (0.40, 0.52)		< 0.001	
Full Scale IQ	-0.00 (-0.02, 0.01)	0.661	-0.01 (-0.02, 0.00)		0.410	-0.02 (-0.04, 0.01)		0.239	
Age	-0.24 (-0.53, 0.12)	0.282	-0.59 (-0.97, 0.18)		0.092	-0.25 (-0.69, 0.69)		0.547	
Sex	-0.88 (-1.17, -0.44)	< 0.001	-0.97 (-1.28, -0.50)		< 0.001	-1.15 (-1.76, -0.60)		0.002	

### Behavioural Difficulties in the Sample Based on SRS Total T-Score

Children scoring in the range T-Score 76 or higher (n = 12), indicating severe social interaction deficits/strongly suspected ASD, had elevated scores in SDQ, in all scales composing the SDQ Total Difficulties Score. The mean SDQ Total Difficulties Score for this subgroup was 19.3, which is above the Swedish cut-off of 14 and, thus, indicating behavioural difficulties. The children scoring in the range between T-Score 60 and 75 (n = 36), indicating social interaction deficits/possible ASD, had a mean of 14.3, i.e. just above the cut-off (Table 5).

**Table 5:** SDQ results in the sample divided in 3 groups based on SRS Total T-Score

	SRS Total T-Score					
	59 or lower n = 826		60 to 75 n = 36		76 or higher n = 12	
SDQ Scales	Mean	SD	Mean	SD	Mean	SD
Total Difficulties Score	5.2	3.8	14.7	5.1	19.3	5.0
Emotional problems	1.3	1.5	3.9	2.3	4.3	2.3
Conduct problems	1.0	1.2	2.6	1.5	4.2	2.7
Hyperactivity/Inattention	2.3	2.2	5.6	3.1	7.0	2.4
Peer problems	0.5	1.0	2.6	2.1	3.8	2.2
Prosocial behaviour	8.9	1.4	7.7	1.7	6.0	3.0

## Discussion

The study examined the distribution of autistic traits in a sample of children from the general population, as well as the association between intellectual functioning and autistic traits, and autistic traits and behavioural difficulties in this sample. The findings suggest a continuous distribution of autistic traits with 4.1% of the total sample having elevated SRS scores, indicating significant deficits in social interaction, and 1.4% scores in the severe range of SRS strongly associated with clinical diagnosis of ASD. FSIQ was negatively associated with autistic traits. This association was more evident in the upper ends of the autistic traits’ continuum. Autistic traits were associated with behavioural difficulties. Being a girl was associated with less behavioural difficulties.

In line with previous studies [4–7], we found a continuous distribution of autistic traits in our sample. There was no clear point distinguishing the children meeting the SRS Total T-Score cut-offs from those who did not, which is consistent with previous studies indicating the lack of natural cut-offs between children with ASD and children without ASD [4–6]. The distribution of SRS Total T-Scores for both sexes was positively skewed, displaying a tail to the right, similar to the findings of Posserud et al. [4].

Consistent with the findings of the US standardization sample [38], boys in our study had higher mean SRS Total Raw Scores than girls. This difference was, however, less marked in our sample, more in line with the German normative sample data reported by Bölte et al. [44]. Furthermore, similar to Bölte et al. [44], the mean SRS Total Raw Score for both boys and girls was slightly lower in our sample than the scores of the US standardization sample reported in the SRS manual [38]. As proposed by Bölte et al. [44] and Kamio et al. [5], this could be related to sample characteristics, since the US sample contains a proportion of twins [38], and male twins tend to receive higher SRS scores than non-twin [51]. In addition, the US sample includes a broader range of ages (from 4 to 18 years) than our sample. It could be so that milder deficits in social interaction and communicative skills become more evident with increased age, when social demands in daily life get more complex.

In our sample, 1.4% (12 children) had SRS Total T-Scores in the range 76 or higher. SRS is not a stand-alone instrument for diagnosis, but scores in this range are associated with a clinical diagnosis of ASD according to SRS manual [38]. Furthermore, fourteen children in the sample (1.6%) had a clinical diagnosis of ASD. The majority of those (n = 12) had a SRS Total T-Score of 70 or higher. About 4% of the sample had T-Scores between 60 and 75 corresponding to the mild to moderate range of SRS according to the manual. These scores reflect clinically significant social interaction deficits and are described as typical for children with mild ASD, but lower scores in this range may also reflect a subthreshold social impairment that may be compensated for when other conditions are not present [38].

In order to further examine the association between intellectual functioning and autistic traits, we conducted two separate quantile regression analyses. The independent variable in the first model was FSIQ and in the second model the independent variables were the WISC-IV indices. In both models we used age and sex as covariates, and SRS Total T-Score as dependent variable. We found a negative association between FSIQ and autistic traits throughout all the percentiles of the SRS Total T-Score distribution. This was more pronounced for those scoring on the higher percentiles of SRS Total T-Score, i.e., children with more autistic traits meaning that having more autistic traits was associated with decrease in IQ scores. The second regression analysis showed that VCI, measuring verbal comprehension, reasoning and conceptualization, and WMI, measuring verbal working memory, were consistently associated with the SRS Total T-Score throughout all percentiles of the SRS Total T-Score distribution. This means that the more autistic traits, the lower verbal function and verbal working memory. This emphasises the connection between autism and verbal ability [52]. Surprisingly, perceptual reasoning and processing speed (PRI and PSI) had no relation with autistic traits. Decreased processing speed scores have been reported in clinical samples of children with ASD [53–55], and have been suggested to be related to adaptive functioning in preschool children with ASD [55]. These associations between ASD and processing speed have not been found when accounting for inattentive symptoms, suggesting that the association between ASD and processing speed could be explained by inattention or ADHD comorbidity [56], which is often present in ASD.

In the quantile regression analyses that examined the association between intellectual functioning and autistic traits, age was associated with autistic traits around the middle quantile of the SRS Total T-Score distribution. This means that age has the highest effect on those with a median level of autistic traits. For neither the 25 quantile nor the 75 quantile has age any significant effect, hence the effect is either too small to be statistically significant or non-significant. Sex was associated with having high level of autistic traits both in the model including FSIQ and in the model including the WISC-IV indices. Contrary to what is expected, being a girl was associated with higher level of autistic traits. However, the difference between sexes was not exaggerated. Furthermore, in the analysis we used SRS sex-based norms, meaning that the effect of sex is already accounted for. The norms are from the US standardisation from about 20 years ago [38]. SRS has now been updated with a newer version (SRS-2) with new norms. This was, however, not published when we performed our study.

In the study, we also wanted to examine the association between autistic traits and behavioural difficulties, and explore the impact of intellectual functioning on this association. The quantile regression analysis showed that autistic traits were positively associated with behavioural difficulties. Higher SRS Total T-Score was associated with higher SDQ Total Difficulties Scores, and this was more marked for the children scoring in the upper ends of the SDQ Total Difficulties Score distribution. These findings are in agreement with previous studies showing associations between autistic traits and scales included in SDQ [34, 35]. In addition, the group of children scoring above the SRS total T-score cut-offs, had means above the SDQ difficulties cut-off. Our findings suggest that an increase in autistic traits increases the risk for additional difficulties in the general population. In contrast, FSIQ was not associated with behavioural difficulties. Although ID (IQ < 70) is a risk factor for behavioural and emotional problems [57], intellectual functioning, according to our study, is not associated with behavioural difficulties in the general population of children.

### Strengths and Limitations

The strength of the study is the large sample size from the general population and with an equal proportion of boys and girls, including a broad range of IQ scores and autistic traits. Furthermore, the study includes well-validated instruments used internationally and assessment by experienced psychologists. The SRS is a quantitative instrument for autistic traits enabling measurement across the complete range of autistic traits and, thus, is suitable for the purposes of the study. The full SRS displayed high internal consistency in our sample, which supports the reliability of this measure in this study. The SDQ Total Difficulties also displayed good reliability.

A limitation of the study is that the assessment of autistic traits was based only on parental report and not included teachers’ report or clinical evaluation. The main limitation of the SRS is that it is not a diagnostic instrument, so scores above cut-off are not equal to clinical diagnosis. Diagnosing ASD requires a comprehensive assessment in multidisciplinary team [58], but instruments such as the SRS can aid the diagnostic process. A significant limitation as regards the measurement of behavioural difficulties is the use of a screening instrument instead of a more comprehensive measure. This enables analysis on general level but not on the relationship between autistic traits and specific areas of behavioural difficulties.

### Clinical Implications

The results highlight the need for school psychologists and teachers to be alert to elevated level of autistic traits and be aware of that intellectual functioning could camouflage these traits.

Particularly lower verbal function and verbal working memory should be noticed. Children displaying a greater degree of autistic traits meeting full criteria of ASD may need additional support, e.g., in school.

## Conclusions

Autistic traits are continuously distributed, with a proportion of children presenting with a greater degree of autistic traits. Intellectual functioning, particularly verbal reasoning and verbal working memory are associated with autistic traits but not with behavioural difficulties. The latter is associated with autistic traits.

### Summary

In this study we aimed to describe the distribution of autistic traits in a cross-sectional sample of children from the general population. We also aimed to examine if intellectual functioning is associated with autistic traits, and if autistic traits are associated with behavioural difficulties. The Swedish Environmental Longitudinal Mother and Child, Asthma and Allergy (SELMA) study is a prospective pregnancy cohort study from the general population in Värmland County, Sweden. Our study includes 874 children (444 boys, 430 girls) from SELMA that were assessed at age 7–9 years with the Wechsler Intelligence Scale for Children – Fourth edition, the Social Responsiveness Scale (SRS), and the Strengths and Difficulties Questionnaire to obtain data on intellectual functioning, autistic traits and behavioural difficulties. We found a continuous distribution of autistic traits. In the sample, 4.1% had elevated SRS scores and 1.4% had scores in the severe range of SRS strongly associated with clinical diagnosis of ASD. We examined the associations between intellectual functioning and autistic traits, and autistic traits and behavioural difficulties using quantile regression, to obtain a comprehensive analysis of the associations throughout the continuum of autistic traits and behavioural difficulties. Intellectual functioning, particularly verbal reasoning and verbal working memory, was negatively associated with autistic traits but not with behavioural difficulties. Behavioural difficulties were associated with autistic traits.

## Data Availability

Not applicable.
